# Regulation of Mitochondrial Morphogenesis by Annexin A6

**DOI:** 10.1371/journal.pone.0053774

**Published:** 2013-01-14

**Authors:** Marcin Chlystun, Michelangelo Campanella, Ah-Lai Law, Michael R. Duchen, Lux Fatimathas, Tim P. Levine, Volker Gerke, Stephen E. Moss

**Affiliations:** 1 Department of Cell Biology, University College London (UCL) Institute of Ophthalmology, London, United Kingdom; 2 Department of Comparative Biomedical Sciences, The Royal Veterinary College, London, United Kingdom; 3 Department of Cell and Developmental Biology, Mitochondrial Biology Group, University College London, London, United Kingdom; 4 University of Muenster, Institute of Medical Biochemistry, Muenster, Germany; 5 Consortium for Mitochondrial Research (CfMR), University College London, London, United Kingdom; University of Florida, United States of America

## Abstract

Mitochondrial homeostasis is critical in meeting cellular energy demands, shaping calcium signals and determining susceptibility to apoptosis. Here we report a role for anxA6 in the regulation of mitochondrial morphogenesis, and show that in cells lacking anxA6 mitochondria are fragmented, respiration is impaired and mitochondrial membrane potential is reduced. In fibroblasts from *AnxA6* ^−/−^ mice, mitochondrial Ca^2+^ uptake is reduced and cytosolic Ca^2+^ transients are elevated. These observations led us to investigate possible interactions between anxA6 and proteins with roles in mitochondrial fusion and fission. We found that anxA6 associates with Drp1 and that mitochondrial fragmentation in *AnxA6* ^−/−^ fibroblasts was prevented by the Drp1 inhibitor mdivi-1. In normal cells elevation of intracellular Ca^2+^ disrupted the interaction between anxA6 and Drp1, displacing anxA6 to the plasma membrane and promoting mitochondrial fission. Our results suggest that anxA6 inhibits Drp1 activity, and that Ca^2+^-binding to anxA6 relieves this inhibition to permit Drp1-mediated mitochondrial fission.

## Introduction

The annexins constitute a family of Ca^2+^ binding proteins with diverse intracellular and extracellular functions that include roles in vesicle transport [1] and inflammation [2]. Annexin A6 (anxA6) has been shown to have a range of activities in cultured cell lines, including the modulation of intracellular Ca^2+^-signalling in epithelial cells [3], Ras inactivation via membrane targeting of p120^GAP^ [4], and the movement of cholesterol from late endosomes to the Golgi and plasma membrane [5]. However, mice containing a homozygous deletion of the *AnxA6* gene do not exhibit any overt phenotype, have a normal lifespan and are fertile [6], indicating that either some of the proposed functions of anxA6 are restricted to cultured cells, or that compensatory mechanisms mitigate the loss of anxA6 in vivo. Despite having a mild phenotype, we have shown that cardiomyocytes from the *AnxA6^−/−^* mice are more contractile and exhibit an accelerated removal of diastolic Ca^2+^ from the cytoplasm [7]. These observations suggest a negative inotropic role for cardiac anxA6, and are consistent with transgenic studies in which cardiomyocyte-specific over-expression of anxA6 led to a reduction in the amplitude of Ca^2+^ transients, impaired contractility and cardiomyopathy [8].

Although the mechanism whereby anxA6 modulates Ca^2+^ signals is not understood, there are many reports of annexins acting both as regulators of cytosolic Ca^2+^ fluxes and also as Ca^2+^ channels themselves [9], [10]. Whilst evidence for the latter is derived solely from experiments using artificial lipid membranes [11], it is clear that annexins can influence Ca^2+^ signals in cells in various ways, including trafficking of channels to the plasma membrane [12], modulation of the cytoskeleton [13] and by direct physical interaction [14]. Here we sought further insight into the role of anxA6 as a regulator of Ca^2+^ signalling, and observed marked differences in mitochondrial ultrastructure in cells and tissues from control and *AnxA6^−/−^* mice. Spatial organisation of the mitochondrial reticulum is crucial for Ca^2+^ homeostasis and influences cellular susceptibility to apoptosis [15]–[17]. We find that mitochondrial morphology is abnormal in cells lacking anxA6, Ca^2+^ signalling and respiration are impaired, and cells have increased resistance to Ca^2+^-mediated apoptosis. Further, we show that a pool of anxA6 associated with mitochondria binds to and inhibits the fission GTPase Drp1, and that elevation of intracellular Ca^2+^ relieves this inhibition by targeting anxA6 to the plasma membrane. Our studies reveal a new function for anxA6, and a novel mechanism of Ca^2+^-dependent regulation of Drp1.

## Results

### Mitochondrial Fragmentation in Cells Lacking AnxA6

**Figure 1:**
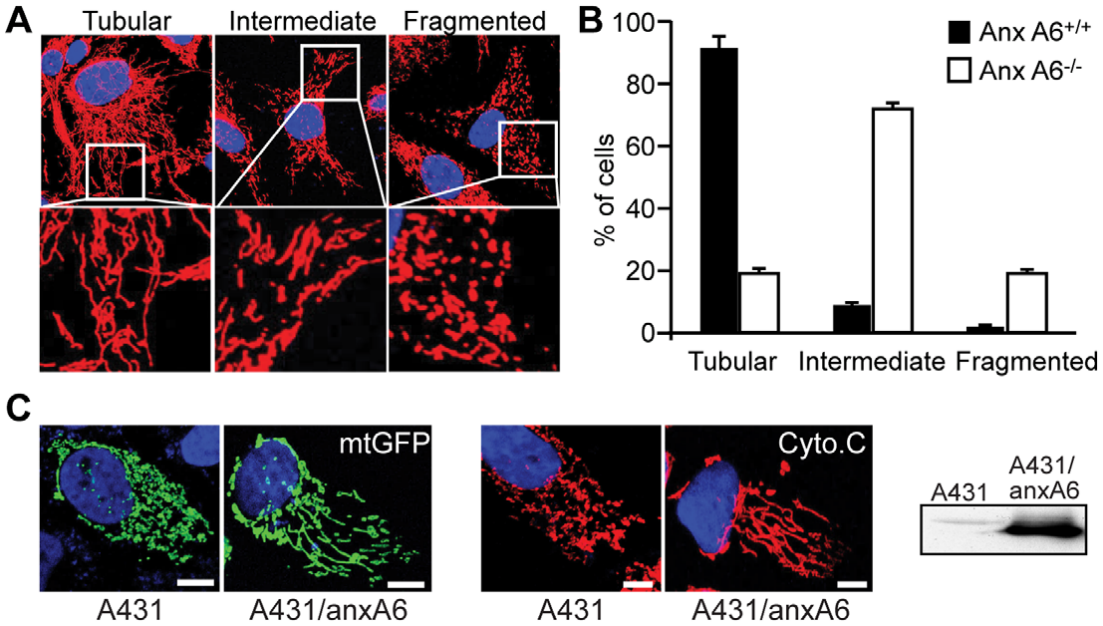
Mitochondrial morphology is abnormal in anxA6 null cells. (A) Images of primary fibroblasts from *AnxA6* ^−/−^ mice immunostained for cytochrome c and analysed by confocal microscopy to visualise mitochondria. (B) Mitochondrial morphology was graded as tubular, intermediate or fragmented for *AnxA6* ^−/−^ and control mice. Data represent the mean ± s.d. of 3 independent experiments, with >1000 cells counted for each strain. (C) A431 cells were either labelled in vivo with mitochondrial-targeted GFP (mtGFP), or labelled following fixation with antibodies to Cyto-c. Cells were counter-stained using DAPI and visualised by confocal microscopy. Representative images show that mitochondrial staining is fragmented in control A431 cells, but tubulated in A431 cells stably expressing AnxA6. Scale bar = 2 µm. The western blot shows expression of the larger isoform of anxA6 only in the stable transfected cells.

**Figure 2:**
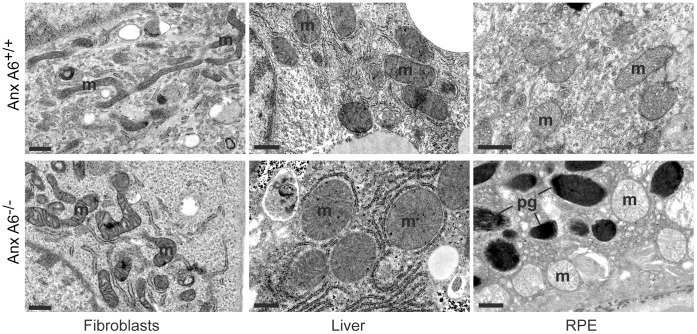
Mitochondrial structural abnormalities in *AnxA6* ^−/−^ mice. Sections of skin, liver and retina were prepared from control and *AnxA6* ^−/−^ mice and examined by electron microscopy. Mitochondria (m) were enlarged and rounded in all tissues, and in retinal pigment epithelial cells (RPE) appeared less electron-dense. Pigment granules in the RPE are also indicated (pg). Scale bar = 500 nm.

Since mitochondria have a critical role in shaping Ca^2+^ signals [18] we examined mitochondrial morphology and function in primary ear fibroblasts from *AnxA6^−/−^* mice and control littermates, and A431 epithelial carcinoma cells (since this cell line does not express anxA6 [3]). Cells were initially labelled either with Mitotracker Red, mitochondrial-targeted GFP (mtGFP), or immunostained with antibodies to the mitochondrial marker cytochrome *c* (Cyto*-c*). Mitochondria were then scored visually according to morphology as tubular/elongated, intermediate or fragmented. In control mice, 89±4.4% of ear fibroblasts exhibited tubular/elongated mitochondria with only a few cells containing fragmented mitochondria. In contrast, only 19±2.7% of *AnxA6^−/−^* cells had tubular mitochondria, almost 20% were fragmented, while the majority displayed an intermediate phenotype (Figure 1, A and B). Similar results were observed in A431 cells using both mtGFP and immunostaining with antibodies to Cyto-*c* to visualise mitochondria (Figure 1C). Western blot analysis confirmed the expression of anxA6 in the stable line, and the absence of anxA6 in the control-transfected wild type cells. The faint band migrating slightly slower than anxA6 in the control cells is most likely weak cross-reactivity with the anxA1 dimer. Thus, wild-type A431 cells lacking anxA6 exhibited mostly fragmented mitochondria whereas ectopic expression of anxA6 in these cells led to the appearance of a tubular mitochondrial reticulum. The effects of anxA6 on mitochondrial morphology were not an artefact of cell culture *ex vivo*, since electron microscopy of mitochondria in skin fibroblasts, liver and retinal pigment epithelial (RPE) cells from the *AnxA6^−/−^* mice showed a rounder, less elongated form, and in RPE cells the mitochondria were also less electron-dense (Figure 2).

**Figure 3:**
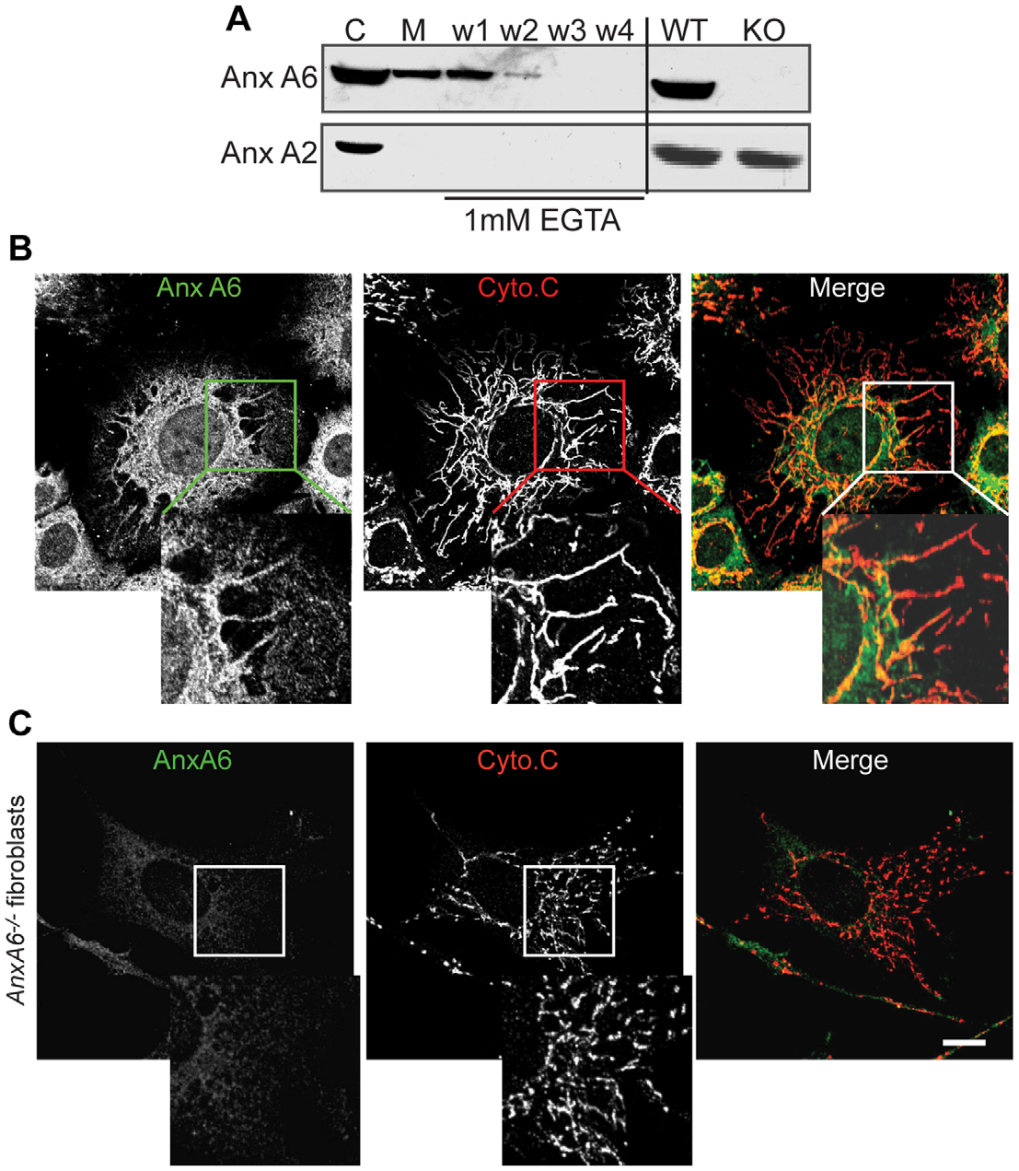
Partial co-localisation of AnxA6 to mitochondria. (A) Mitochondria (M) and cytosol (C) were purified from normal mouse liver and western blotted for AnxA6 and AnxA2. The mitochondrial samples were immunoblotted following repeated washes (lanes w1-w4 show protein in each wash) in buffer containing 1 mM EGTA, as were whole liver cell extracts from control (WT) and *AnxA6* ^−/−^ (KO) mice. (B) Normal primary mouse fibroblasts were co-immunostained for AnxA6 and cytochrome C and analysed by confocal microscopy. Insets show partial co-localisation of AnxA6 and Cyto-c on mitochondria. (C) Primary mouse fibroblasts were isolated from the ears of *AnxA6* ^−/−^ mice and fixed and immunostained for AnxA6 and Cyto-c The images show faint non-specific staining with the AnxA6 antibody, and the characteristic pattern of fragmented mitochondria (see inset). Scale bar = 4 µm.

To determine how the subcellular localisation of anxA6 related to mitochondrial morphology, we performed western blotting and confocal microscopic analysis of purified mouse liver mitochondria and primary mouse fibroblasts respectively. Most annexins are predominantly soluble and cytosolic [10]. Consistent with this, immunoblotting revealed both anxA6 and anxA2 in cytosolic fractions purified from mouse liver. However, only anxA6 was also present in the mitochondrial fraction, demonstrating that the mitochondrial association of anxA6 is specific and not a generic annexin property. Moreover, anxA6 was retained in the mitochondrial fraction following exhaustive washing with the Ca^2+^ chelator EGTA (Figure 3A), revealing the interaction with this organelle to be Ca^2+^-independent. The association of anxA6 with mitochondria was further confirmed by confocal microscopy, which identified co-localisation with Cyto*-c* in control mouse ear fibroblasts (Figure 3B). The specificity of the anxA6 antibody was verified by the absence of staining in *AnxA6* ^−/−^ mouse ear fibroblasts (Figure 3C).

### Loss of AnxA6 Disrupts Ca^2+^-signaling and Mitochondrial Bioenergetics

**Figure 4:**
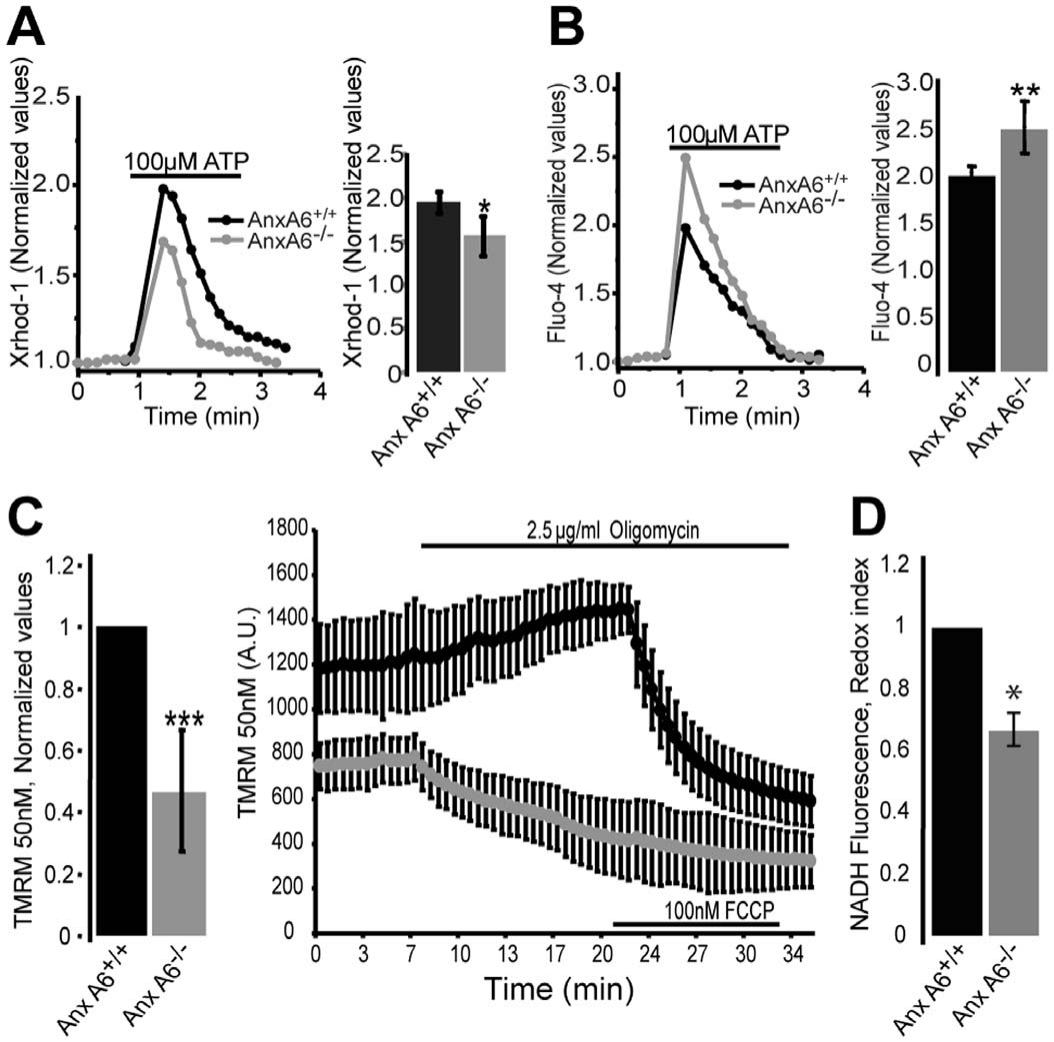
Ca^2+^ signalling and mitochondrial physiology defects in cells lacking anxA6. (A) Cytosolic Ca^2+^ transients in *AnxA6* ^+/+^ and *AnxA6* ^−/−^ fibroblasts in response to ATP measured with Fluo-4. The pooled data are shown in the adjacent histograms comparing peak responses. (B) Representative traces of mitochondrial Ca^2+^ uptake in *AnxA6* ^+/+^ and *AnxA6* ^−/−^ fibroblasts with histogram summarizing relative values assessed with XRhod-1 in response to stimulation with the IP3 generating stimulus ATP (100 mM) in both cell types. (C) ΔΨm in *AnxA6* ^+/+^ and *AnxA6* ^−/−^ ear fibroblasts was measured in cells equilibrated with 50 nM TMRM under resting conditions. The TMRM intensity, normalized to that of the *AnxA6* ^+/+^ cells demonstrates a reduction in potential in *AnxA6* ^−/−^ cells. In response to oligomycin, ΔΨm increased in *AnxA6* ^+/+^ cells but decreases in the *AnxA6* ^−/−^ cells, suggesting that ΔΨm is maintained by a reversal of the F1-FoATPsynthase. (D) NADH autofluorescence was measured and consecutive additions of NaCN and FCCP were used to establish the dynamic range of the signal and to normalize the resting state in relation to the maximally reduced and maximally oxidised signals (respectively). The normalized values show that the NADH redox state was more oxidized in *AnxA6* ^−/−^ cells consistent with a degree of uncoupling.

To determine whether the mitochondrial fragmentation seen in *AnxA6* ^−/−^ fibroblasts affected intracellular Ca^2+^ homeostasis and signaling we examined mitochondrial Ca^2+^ uptake and cytosolic Ca^2+^ transients following stimulation of control and *AnxA6* ^−/−^ fibroblasts with ATP (Figure 4, A and B). To this end, cells were loaded with the fluorescent dyes Xrhod-1 and Fluo-4 AM to concomitantly monitor Ca^2+^ signals in the two compartments. The traces and the plotted peak values show that mitochondrial [Ca^2+^] transients were reduced in *AnxA6* ^−/−^ cells (grey trace) compared to control cells (black trace) - the relative increase in Xrhod signal in response to ATP fell from 1.87±0.12 in *AnxA6^+/+^* cells to 1.56±0.22 in the *AnxA6^−/−^* cells * = p<0.05, n = 3). In contrast, cytosolic [Ca^2+^] transients were increased in *AnxA6^−/−^* cells compared to controls, such that the relative Fluo-4 peak response rose from 1.97±0.17 in *AnxA6^+/+^* cells to 2.41±0.25 in the *AnxA6^−/−^* cells **p<0.005, n = 4). Therefore the decreased mitochondrial Ca^2+^ uptake reflects a specific mitochondrial defect, and not a global decrease in Ca^2+^ signalling.

Measurements of mitochondrial membrane potential (ΔΨ_m_) using the potentiometric dye TMRM revealed that ΔΨ_m_ was significantly reduced in *AnxA6^−/−^* cells with a 54% decrease in TMRM fluorescence signal (Figure 4C) *** = p<0.001, n = 36). The reduced ΔΨ_m_ may account for the attenuated mitochondrial [Ca^2+^] uptake in *AnxA6^−/−^* cells, reflecting a decreased driving force for Ca^2+^ accumulation [19]. Inhibition of the F_1_-FoATP synthase using oligomycin, caused a loss of ΔΨ_m_ in the *AnxA6* ^−/−^ cells rather than the expected increase in potential seen in control cells (Figure 4C), showing that in the *AnxA6* ^−/−^ cells ΔΨ_m_ is maintained by the reversal of the ATP synthase which operates as a proton pumping ATPase. This suggests that loss of AnxA6 causes a bioenergetic defect – such that the ΔΨ_m_ is sustained instead by the activity of the ATPase [20], [21]. To further investigate the change in mitochondrial physiology, NADH autofluorescence was measured as an index of mitochondrial redox state. NaCN and FCCP were employed to establish the full dynamic range of the NADH fluorescence signal as the maximally reduced and maximally oxidized signals respectively. The resting level was defined in relation to these signals, thus indicating the resting state of the NAD^+^/NADH pool which we will refer to as ‘redox index’ (Figure 4D). The NADH/NAD^+^ redox state was significantly more oxidized in *AnxA6* ^−/−^ cells than the controls (*AnxA6^+/+^* cells: 1; *AnxA6* ^−/−^ cells: 0.65±0.051, * = p<0.05, n = 39). The combination of a reduced potential and a more oxidised redox state strongly suggests a degree of uncoupling in cells lacking *AnxA6.*

**Figure 5:**
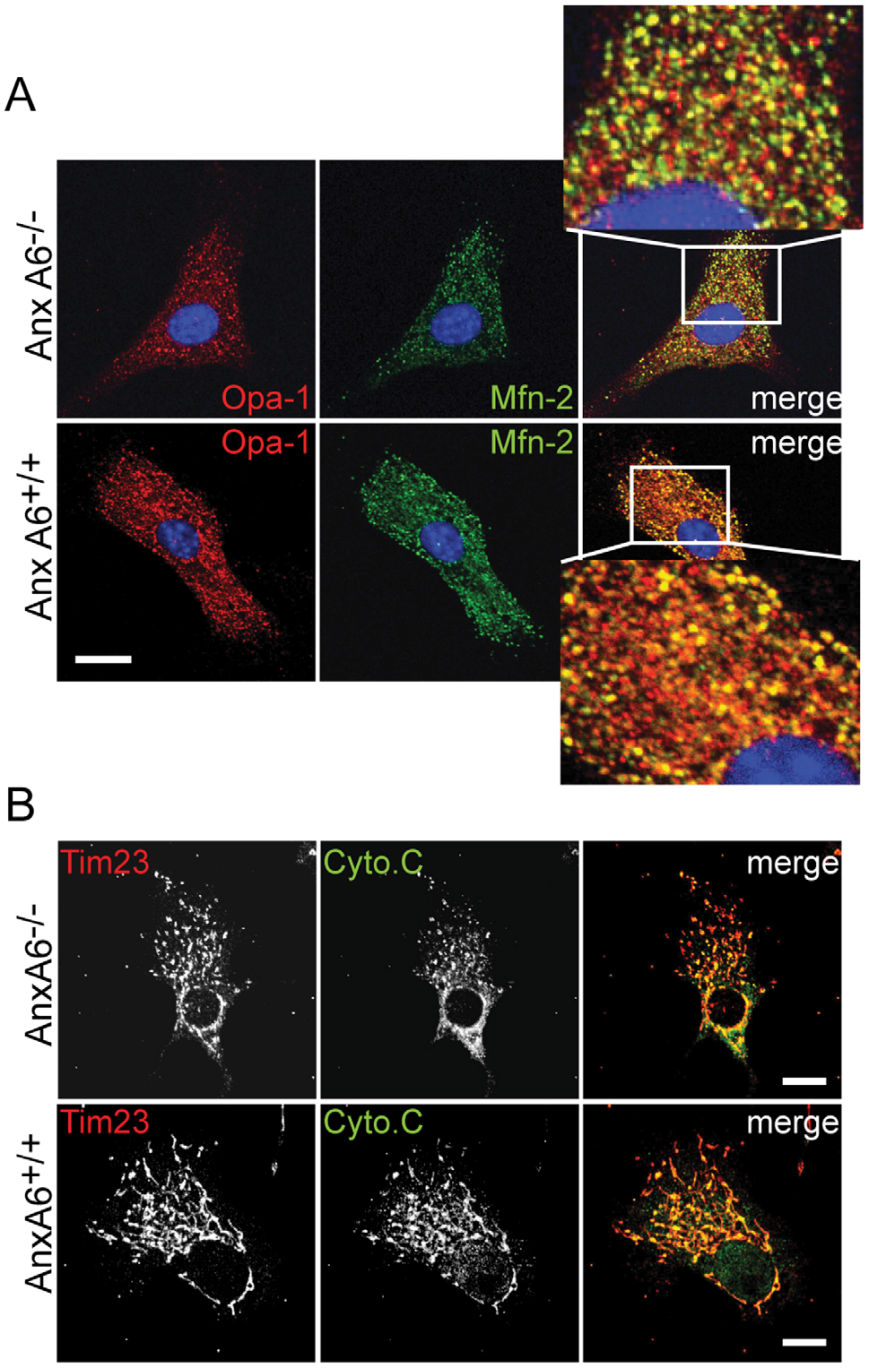
Loss of mitochondria-ER contact sites in *AnxA6* ^−/−^ cells. (A) Opa-1 and Mfn-2 co-localization in *AnxA6^+/+^ and AnxA6^−/−^* fibroblasts mark the overlap between ER and mitochondria in the two cohorts of cells. Scale bar = 4 µm. (B) Primary mouse fibroblasts fixed and immunostained for Tim23 and Cyto-c The images show co-localisation of Tim23 and Cyto-C in fibroblasts from both mutant and wild type mice, but with the characteristic mitochondrial fragmentation in AnxA6 null cells. Scale bar = 5 µm.

Close apposition between ER and mitochondria is critical for the efficient exchange of Ca^2+^ between these compartments following elevation of intracellular Ca^2+^ mediated by InsP_3_ [18], [22]. To find out whether the anomalous patterns of Ca^2+^ signalling observed in cells lacking anxA6 could be due to changes in ER-mitochondria connectivity we used immunofluorescence to examine the distribution of Mfn2 [23], which localises to mitochondria-ER contact sites [24]. We compared this with the staining pattern of the Optical Atrophy 1 (Opa 1) protein which is involved in the folding of the inner mitochondrial cristae [25] and thus represents a suitable readout for the structural integrity of the mitochondrial network (Figure 5A). Quantitative image analysis revealed significantly reduced co-localisation of these two proteins in *AnxA6* ^−/−^ fibroblasts than control cells, suggesting a decrease in ER-mitochondria contact sites (*AnxA6^+/+^*: 90.28±8.36%; *AnxA6^−/−^*: 75.78±8.20%; n = 2 cells). The loss of ER-mitochondria contact sites was not the consequence of a non-specific loss of mitochondrial integrity, as the localisation of Tim23 was unaffected in anxA6 null cells (Figure 5B). The loss of mitochondria-ER contact sites is consistent with the observation that mitochondrial Ca^2+^ uptake is defective in *AnxA6* ^−/−^ fibroblasts, and may also explain our previous report that EGF-induced Ca^2+^ transients in A431 cells are attenuated by ectopic expression of anxA6 [3].

### AnxA6 Modulates Cellular Susceptibility to Apoptosis

**Figure 6:**
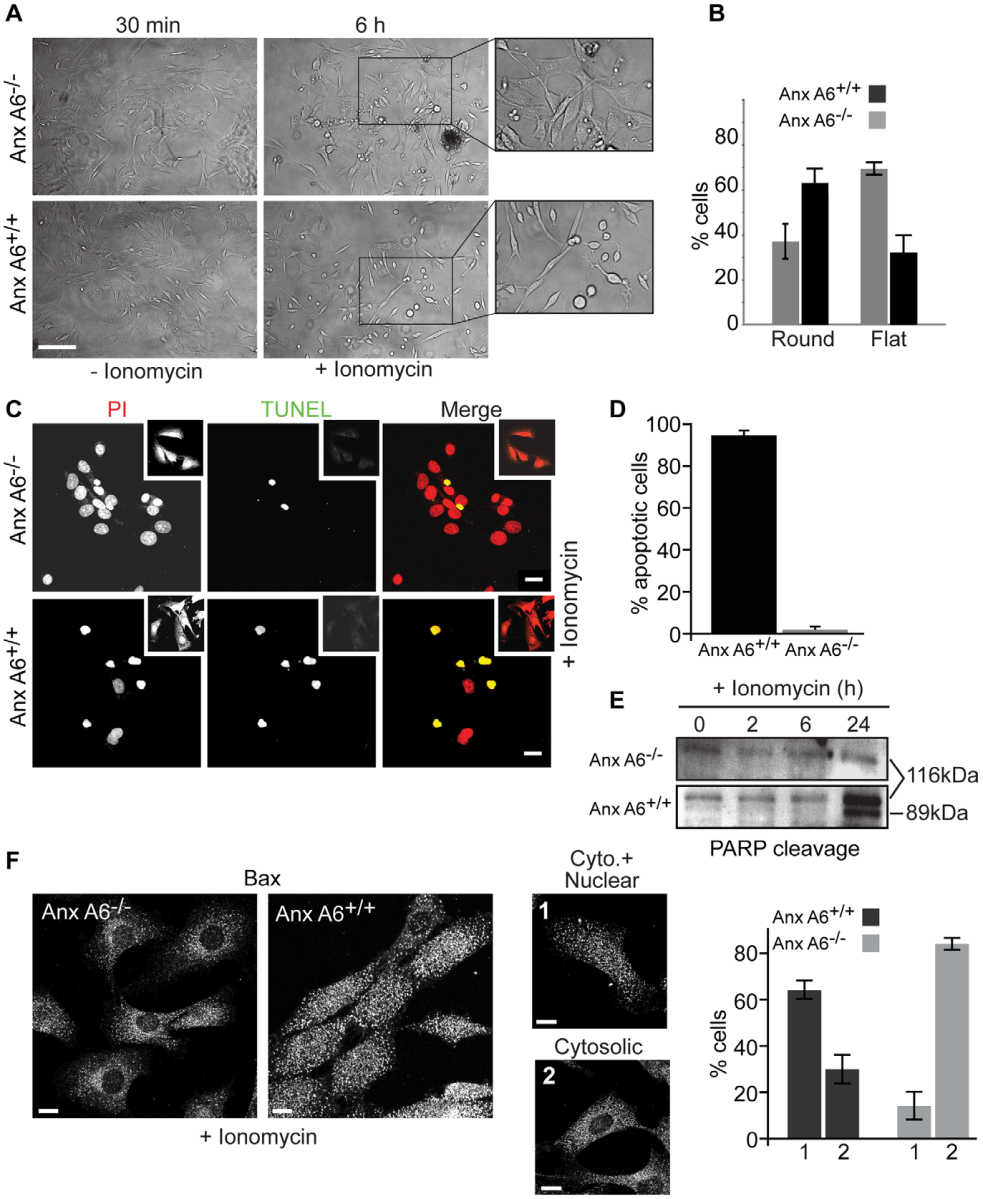
AnxA6 null cells are resistant to Ca^2+^-induced apoptosis. (A) Primary mouse fibroblasts were isolated from the ears of control and *AnxA6* ^−/−^ mice and exposed to 1 µM ionomycin for the times indicated. Phase contrast images (and insets) show rounding up of cells after 6 h. Scale bar = 30 µm. (B) Quantification of round and flat cells (>1000 in 3 separate experiments). (C, D) Fibroblasts from control and *AnxA6* ^−/−^ mice were incubated with 1 µm ionomycin for 6 h, then either labelled with propidium iodide (PI) and TUNEL stained for confocal imaging (C) or sorted by flow cytometry (D) to quantify the number of apoptotic cells. Scale bar in C = 10 µm. (E) Lysates were prepared from cells as above, after various times in ionomycin, and western blotted for PARP. The full-length protein (116 kDa) and cleavage product (89 kDa) are indicated. (F) Control and AnxA6 null fibroblasts were exposed to ionomycin as in (C) then fixed and stained for Bax. Staining for Bax was either both nuclear and cytosolic (image 1) or cytosolic (image 2). Cell populations (n ≥1300 cells in three separate experiments) were scored for each pattern of staining and the percentage of cells displaying each phenotype were plotted in the histogram. Scale bar = 5 µm.

Since defects in mitochondrial Ca^2+^ handling would also be expected to influence cellular susceptibility to apoptosis, we next examined the effects of loss of anxA6 on the response of primary mouse ear fibroblasts to the Ca^2+^ ionophore ionomycin. TUNEL staining, PARP cleavage and nuclear translocation of Bax were used as read-outs for apoptosis. After 24 h in the presence of 1 µM ionomycin a significant number of control cells became rounded, whereas anxA6 null fibroblasts appeared morphologically normal (Figure 6, A and B). Fluorescence microscopy revealed fewer TUNEL positive anxA6 null cells compared to control cells following ionomycin treatment (Figure 6C). This was confirmed by quantification by flow cytometry, which showed that 90±7.4% control cells were positive on TUNEL staining, whereas fewer than 5% of *AnxA6* ^−/−^ fibroblasts were positive (Figure 6D). Consistent with these observations, western blotting of cells treated as above showed marked PARP cleavage [26] in control but not in *AnxA6* ^−/−^ fibroblasts (Figure 6E). Nuclear translocation of Bax, concomitant with that towards mitochondria, has also been reported in cells undergoing apoptosis [27]. Here, we observed that >60% of control cells exhibited nuclear Bax, compared to <30% of *AnxA6* ^−/−^ fibroblasts following ionomycin stimulation (Figure 6F). Collectively these data show that when assessed using a variety of read-outs, loss of anxA6 leads to increased resistance to Ca^2+^-dependent apoptosis. This may be relevant in certain cancers where loss of AnxA6 correlates with tumorigenesis and a more malignant phenotype [28], [29].

### AnxA6 Binds to Drp1

**Figure 7:**
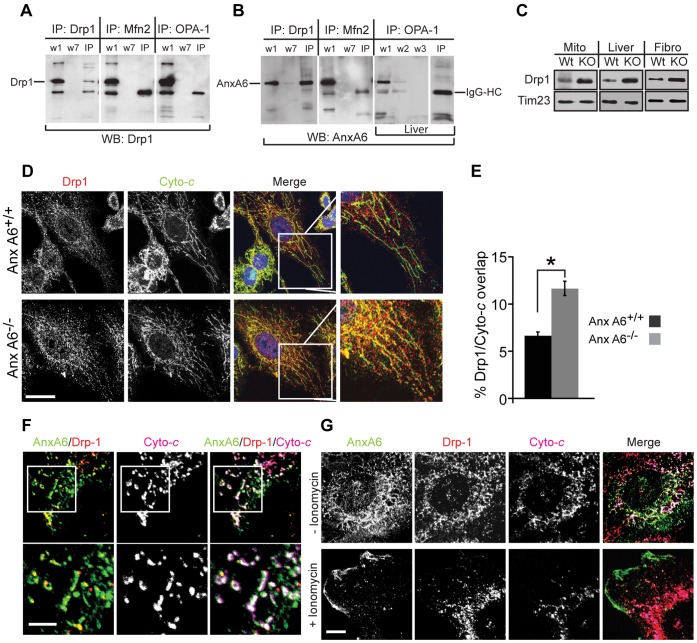
AnxA6 regulates mitochondrial morphology via interaction with Drp1. (A, B) Whole cell lysates were prepared from control mouse ear fibroblasts or liver) and immunoprecipitated (IP) with antibodies to Drp1, Mfn2 and OPA1 as indicated. For each blot, w1 corresponds to input, and w2, w3 and w7 correspond to wash number. IP is the immunoprecipitate, and the position of AnxA6 is indicated. Blots were probed with antisera to Drp1 (A) and AnxA6 (B), the position of which is indicated, and visualised using enhanced chemiluminescence. The band running beneath AnxA6 in the Mfn2 and OPA-1 IP lanes is IgG heavy chain. (C) Purified mitochondria and whole cell lysates of liver and primary fibroblasts from control (Wt) and *AnxA6* ^−/−^ (KO) mice were immunoblotted with antisera against Drp1 and the mitochondrial marker Tim23 as a control for loading. (D) Fibroblasts from control and *AnxA6* ^−/−^ mice were immunostained with antisera to Drp1 (red) and the mitochondrial marker cytochrome C (green), and analysed by confocal microscopy. The insets show reticular staining of Cyto C in control fibroblasts, with little co-localisation with Drp1 (visualised in orange), in contrast to increased co-localisation of the two proteins in the AnxA6 null cells. Scale bar = 5 µm. (E) The proportion of Drp1 immunofluorescence coincident with Cyto-c was calculated using Metamorph, and is presented as mean ± s.d., n = 3000 cells in 3 separate experiments, *p<0.05. (F) A431 cells stably expressing AnxA6 were triple stained for AnxA6 (green), Drp1 (red) and Cytochrome c (magenta). Regions that appear white in the lower right zoomed panel indicate coincidence of the three antigens. Scale bar = 2 µm. (G) A431 cells stably expressing AnxA6 were simulated with 1 µm ionomycin for 5 min, then fixed and stained for AnxA6, Drp1 and Cytochrome c as in (F). Note that AnxA6 relocates from the cytosol to plasma membrane in cells exposed to ionomycin (lower right ‘merge’ panel), with loss of regions of coincident staining of the three proteins (seen as white in the top right ‘merge’ panel). Scale bar = 5 µm.

Mitochondrial fragmentation is frequently observed at the onset of apoptosis [30], and has thus become associated with increased susceptibility to apoptosis. Although this appears to run contrary to the data shown in Figure 6, there are exceptions to this paradigm [31]. In particular, mitochondrial fission induced by over-expression or activation of Drp1 leads specifically to resistance to Ca^2+^-dependent apoptosis [32]. Interestingly, such manipulation of Drp1 mediates the opposite effect with regard to apoptosis triggered by stimuli acting through other pathways [32]–[34]. This prompted us to examine whether the fragmentation of mitochondria in cells lacking anxA6 is due to an imbalance in the large GTPases, in particular Mfn2, Drp1 and Opa1. To determine whether anxA6 exerts its influence on mitochondrial morphogenesis via a physical interaction with any of these molecules, we performed co-immunoprecipitation experiments with antisera to Drp1, Mfn2 and Opa1, followed by western blotting with antisera to anxA6 (Figure 7, A and B). Immunoprecipitates (IPs) were conducted using cell lysates prepared from both mouse ear fibroblasts and mouse liver. First, we looked for evidence of interactions between Drp1 and the other two GTPases. In these experiments we used Drp1 antibodies to immunoblot the first pellet wash (w1), the final pellet wash (w7– to ensure no detectable protein carried through), and the IP (Figure 7A). Drp1 was detected in each case in the first but not in the final wash, and only in the Drp1 IP, showing that Drp1 does not interact with either Mfn2 or Opa1. Next, using a similar protocol we immunoblotted Drp1, Mfn2 and Opa1 IPs for anxA6 (Figure 7B). These experiments revealed the presence of anxA6 in Drp1 IPs but not in those for Mfn2 or Opa1, showing that anxA6 forms a physical association with Drp1.

We then examined the expression and mitochondrial targeting of Drp1 in cells lacking anxA6, and observed both increased expression of Drp1 in whole cell lysates, and increased association of Drp1 with purified liver mitochondria from *AnxA6* ^−/−^ mice (Figure 7C). In contrast the mitochondrial protein Tim23 was expressed at the same level in all samples tested, suggesting that absolute mitochondrial load is apparently unchanged as a consequence of *AnxA6* gene knock-out. When control and anxA6 null fibroblasts were co-stained for Drp1 and Cyto-*c*, we observed a significantly higher level of Drp1 immunoreactivity localised to mitochondria in *AnxA6* ^−/−^ fibroblasts (Figure 7, D and E). These observations suggest that anxA6 inhibits the targeting of Drp1 to mitochondria, and that in cells lacking anxA6 Drp1 associates with mitochondria and drives fission. In A431 cells stably expressing anxA6 we observed marked co-localisation of anxA6 and Cyto-*c*, consistent with data in Figure 3B, with Drp1 staining appearing punctate at sites of mitochondrial association (Figure 7F). Stimulation of these cells with ionomycin led to translocation of anxA6 from intracellular sites, including mitochondria, to the plasma membrane (Figure 7G). The behaviour of anxA6 in this context is in line with the general paradigm that annexins become membrane-associated upon elevation of intracellular Ca^2+^ [1], [10]. Numerous studies have described this phenomenon, which is due to the specific affinity of annexins for negatively-charged phospholipid headgroups in the presence of Ca^2+^ [13], [35], [36].

**Figure 8:**
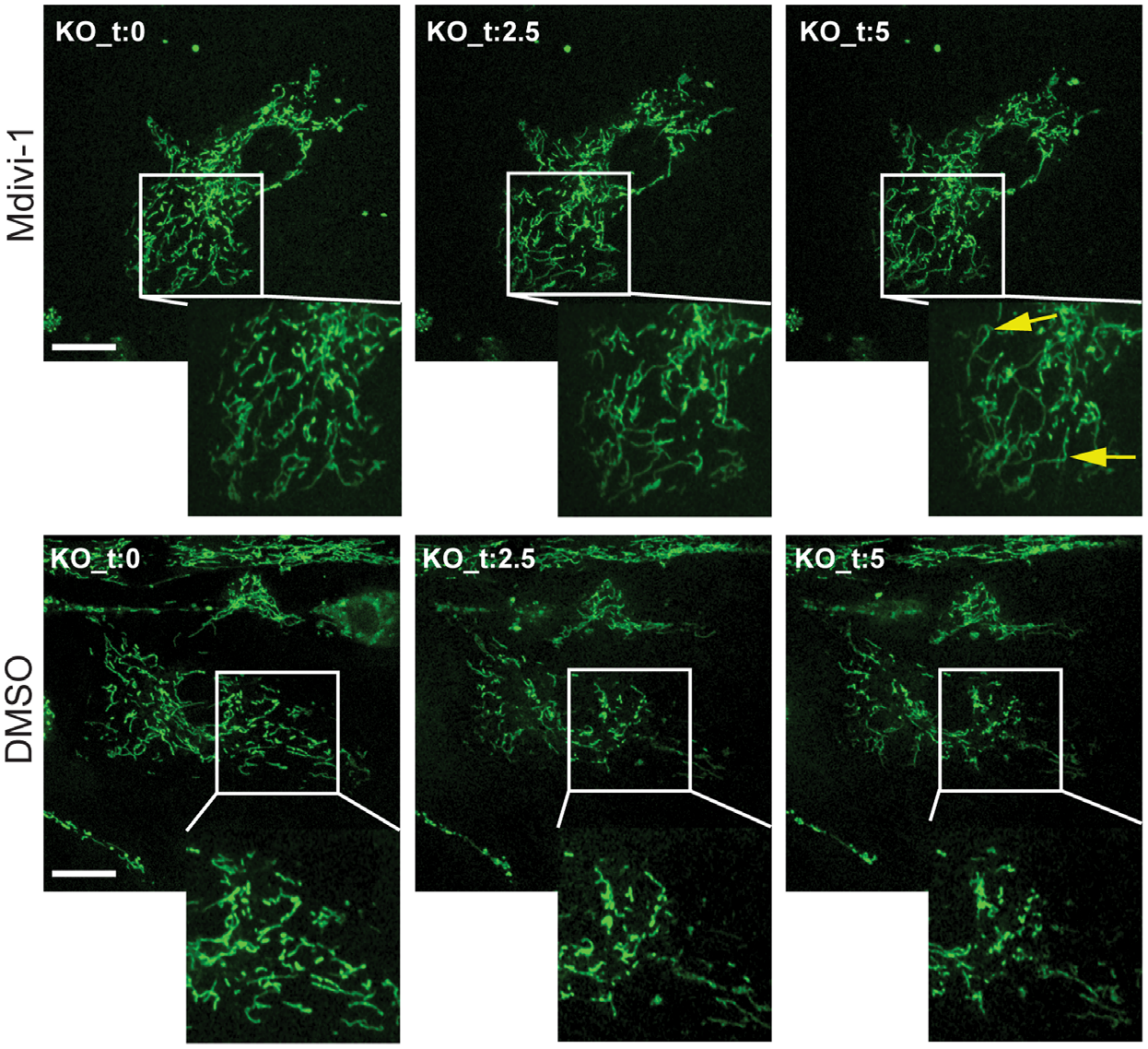
Inhibition of Drp1 reverses mitochondrial fragmentation in AnxA6 null fibroblasts. Primary mouse fibroblasts were isolated from the ears of *AnxA6* ^−/−^ mice, loaded with Mitotracker, and exposed to DMSO (control) or 50 µM Mdivi-1 in DMSO for up to 15 min. Images were captured on an inverted confocal microscope at 0, 2.5 and 5 min. The zoomed regions in the top panels show a marked increase in the number and length of mitochondrial extensions (yellow arrows), in contrast to the DMSO treated cells in which the mitochondria remained mostly fragmented. Scale bar = 5 µm.

These observations suggest that inhibition of Drp1 in anxA6 null fibroblasts should be sufficient to reverse the mitochondrial phenotype in those cells. To test this hypothesis we examined the effects of a small molecule inhibitor of Drp1 named mdivi-1 [37] on primary fibroblasts isolated from the *AnxA6* ^−/−^ mouse (Figure 8). Cells were loaded with mitotracker and imaged at various times upon exposure to mdivi-1. The results show that over a 15 minute period there was a gradual increase in connectivity within the mitochondrial reticulum that was not observed in the DMSO control.

## Discussion

Mitochondrial morphogenesis is under the dynamic control of a large number of proteins, among which Drp1 is considered the master regulator of mitochondrial fission [16]. Drp1 is itself subject to regulation by multiple factors including Ca^2+^ and cAMP, ubiquitylation and several interacting proteins. Elevation of intracellular Ca^2+^ can stimulate phosphorylation of Drp1 on Ser 600 by CaM kinase Iα [38] as well as dephosphorylation of Drp1 on conserved serines 656 and 637 by calcineurin [39], [40]. In each instance Ca^2+^ stimulates translocation of Drp1 to mitochondria and fragmentation. Here we provide evidence for a further mechanistic link between Drp1, Ca^2+^ and mitochondrial network morphogenesis, and suggest a model in which anxA6 constrains mitochondrial fission by Drp1 at physiological Ca^2+^ levels.

The effects of anxA6 gene knock-out on mitochrondrial morphogenesis are in part similar to those recently reported for the novel mitochondrial outer membrane protein MIEF1/MiD51 [41], [42]. As with MIEF1, over-expression of anxA6 in cells normally lacking anxA6 led to extensive mitochondrial fusion, whereas mitochondria in cells lacking anxA6 were highly fragmented (Figure 1). And like MIEF1, anxA6 is not expressed in invertebrates [43], further emphasising the point that mitochondrial morphogenesis is differentially regulated in vertebrates and yeast. There is no evidence that anxA6 is an integral mitochondrial membrane protein, but we identified a pool of anxA6 that is tightly associated with mitochondria, remaining bound even after exhaustive washing with Ca^2+^ chelator. The paradigm for membrane binding by annexins is that it is Ca^2+^-dependent and reversible, but there are well characterised exceptions to this model such as the Ca^2+^-independent binding of anxA2 to membranes enriched in phosphatidylinositol 4,5-bisphosphate [44], [45]. Here we showed that anxA2 is not associated with mitochondria, demonstrating that this characteristic of anxA6 is not a generic annexin property.

Division of the mitochondrial network disturbs the association between mitochondria and intracellular sources of Ca^2+^, and thus reduces mitochondrial Ca^2+^ uptake [32]. Although cell viability isn’t affected in the absence of anxA6 such deficiency (or adaptation) mediates protection against stress responses involving ER/mitochondrial crosstalk and mitochondrial Ca^2+^ overload such as programmed cell death induction via ionomycin. Consistent with this, we observed enhanced resistance to Ca^2+^-dependent apoptosis in anxA6 null fibroblasts, despite extensive fragmentation of the mitochondrial network in these cells. Interestingly, we previously showed that targeted deletion of the *AnxA5* gene in chicken DT40 cells similarly led to resistance to Ca^2+^-dependent apoptosis [46], suggesting that these two annexins may have specific and complementary activities in this context. In cells lacking anxA6 we also observed a significant loss of mitochondrial membrane potential. Given that anxA6 binds to Drp1, we attribute this to deregulated activation of Drp1 since work elsewhere has shown that over-expression of Drp1 in the presence of an inducer of apoptosis inducer such as staurosporine, is sufficient to accelerate the collapse of ΔΨ_m_ [47], [48].

In normal cells it is well established that mobilisation of intracellular Ca^2+^ targets anxA6 to the plasma membrane [1], [49], which we propose here relieves the functional blockade of Drp1 by anxA6 to permit Drp1-mediated mitochondrial fragmentation. Our results using primary cells from *AnxA6* ^−/−^ mice show that loss of the anxA6-Drp1 interaction is sufficient to drive mitochondrial fission irrespective of changes in cytosolic Ca^2+^ concentration, and presumably therefore, the activities of other modulators of Drp1 and the phosphorylation/dephosphorylation cycle mentioned earlier. In this delicate interplay between structure and signalling, anxA6 emerges as an important regulator of the mitochondrial fission factor Drp1 and, through physical interaction with Drp1, may thus be central to shaping Ca^2+^ responses in both normal and diseased cells.

## Materials and Methods

### Cells and Mice

AnxA6 null mutant mice and A431 cell culture techniques have been described previously [3], [6]. All animals were bred and maintained by Biological Services in the UCL Institute of Ophthalmology. The experiments described in this study were carried out under licence from the UK Home Office and following approval from the UCL Institute of Ophthalmology Ethical Review Panel. The primary ear fibroblasts were isolated as follows using two or three month old mice. Animals were killed by cervical dislocation and quickly placed on ice. Ears were removed and washed briefly in ice-cold 70% ethanol, followed by ice-cold PBS. Hair and the epidermal layer of the skin were removed with a medical scalpel and the ears washed once more in fresh ice-cold PBS. Collagenase II (Worthington Biochemical) was prepared at the working concentration of 2.5 mg/ml in serum-free DMEM. Each ear was submerged in 1 ml of the collagenase solution followed by fragmentation into small pieces with a fresh scalpel. Fresh collagenase solution (4 ml) was added and the suspensions resulting from the preparation of two ears were transferred to a small cell culture bottle and incubated for up to 3 h at 37°C with vigorous mixing every 15–30 min. The resulting cell suspension was diluted 3x and transferred to 6–well culture dishes containing 22 mm cover slips treated with 70% ethanol followed by washing in PBS. Each well was filled with 1 ml of diluted cell suspension and 2 ml of fresh DMEM containing 10% FCS. Cells were incubated at 37°C for 24 to 36 h before experimentation. This procedure was applied on a regular basis unless the specific requirements of an experiment dictated otherwise.

### Immunofluorescence and Confocal Microscopy

The rabbit polyclonal antibodies to anxA6 have been described previously [50]. For immunofluorescence studies antibodies were diluted 1∶100 and cells were used according to the following protocol. Cells at 60–70% confluence were washed 2x with ice-cold PBS containing a mixture of 0.1% Tween/0.1% Triton, and then fixed in 3% ice-cold PFA for up to 10 min at room temperature. Cells were washed quickly 2x in PBS, and 0.3% Tween 20 and Triton X-100 was applied for up to 20 min to achieve full permeabilisation of the cell surface as well as intracellular organelles, in particular mitochondria. After permeabilisation, cells were washed 3x in PBS containing 0.1% Tween 20, then briefly exposed to 50 mM NH_4_Cl followed by washing 2x in PBS. Primary antibodies were applied for 2 h at room temperature or overnight at 4°C. Labelled cells were washed up to 5x with PBS containing 0.1% Tween 20 and incubated for up to 1 h at room temperature with secondary antibodies, then washed again up to 10x with PBS containing 0.01% Tween 20. The labelling or co-labelling procedures including antibodies to Cyto-*c* (mono- or poly, 1∶500 and 1∶1000 respectively, Santa Cruz), Drp1 (mono, 1∶500, BD Biosciences), Opa1 (1∶500, BD Biosciences), Tim23 (1∶500, BD Biosciences) and Mfn2 (1∶1000, Santa Cruz) followed a similar procedure with the only difference being that Cytochrome C staining was always done overnight at 4°C and the primary antibodies were never placed on cells simultaneously, but sequentially with 2–3x washes with PBS containing 0.01% Tween 20 in between. The procedure for labelling cells with mitochondria-targeted GFP employed a standard lipofectamine-based protocol as describe elsewhere [51]. Mitotracker Green (Molecular Probes) was applied according to the standard procedure for primary cells. Cells loaded with mitotracker were incubated for 15–30 min at 37°C and washed up to 8x with warm PBS. Immunofluorescence images were obtained on a Zeiss 510 confocal microscope and processed on Zeiss as well as Metamorph software. Live images of mitotracker-labelled cells were obtained on a Leica AOBS confocal microscope in the live-imaging mode and processed on Velocity software. Mitochondria were purified from mouse liver as described elsewhere [52].

### Immunoprecipitation, Western Blotting and SDS-PAGE

The interactions between AnxA6 and other mitochondrial factors were investigated by immunoprecipitation under non-denaturing, [Ca^2+^] plus and [Ca^2+^] minus conditions. Cells and/or organs of interest were lysed and prepared according to standard procedures associated with the DynaBeads protocol. Lysates were always pre-incubated with unconjugated beads for 10 min prior to incubation with antibodies overnight at room temperature. Each washing step was retained and checked on western blots. The elution was cross-checked for the presence of the primary antibody as well as the presence of a putative interacting partner. Standard western blotting procedures were applied following 10% SDS-PAGE. Blots were incubated with antibodies diluted usually at the concentration of 1∶1000 (for primary antibodies) or 1∶5000 (for secondary antibodies) in 10% skimmed milk dissolved in DPBS containing 0.01% Tween 20.

### Apoptosis Measurements

To assess the susceptibility of *AnxA6* knock-out cells to Ca^2+^ - induced apoptosis, cells were exposed to 1 µM ionomycin for up to 24 h and visually inspected for the appearance of a rounded phenotype. To more precisely assess the induction of apoptosis, ionomycin-exposed cells were tested with a standard TUNEL protocol according to the manufacturer’s instructions (Cell Signalling). Propidium iodide was used as contrast agent on permeabilised cells. To further assess apoptosis, PARP labelling was applied. The ionomycin-exposed control and AnxA6 knock-out cells were lysed and samples were analysed by SDS-PAGE and western blotting. Anti-PARP antibodies (Cell Signalling) were used to assess the induction of apoptosis. Bax antibodies (Santa Cruz) were used to study nuclear translocation in conjunction with immunofluorescence labelling of control and *AnxA6* knock-out cells exposed to high concentration of ionomycin. Quantification of signals from immunofluorescence analysis was performed on the microscope by taking each picture 3x and averaging the intensity of signals.

### Mitochondrial Ca^2+^-measurements

Measurement of mitochondrial and cytosolic Ca^2+^ was executed with the fluorescent dyes X-rhod and Fluo-4 AM. Primary fibroblasts were co-incubated with the dyes (5 µM doses) and 0.002% (w/v) pluronic acid for 50 min at 37°C. The solution was then removed and cells were washed twice with modified HBSS. A fresh aliquot of modified HBSS (was added to the cells and each cover slip of cells was mounted on an imaging chamber to be affixed on the stage of the camera imaging system (Zeiss LSM-510). Acquired images were analysed with the LSM and Image-J Software.

### Imaging of Mitochondrial Transmembrane Potential (ΔΨ_m_)

We have conducted this experiment using the dye tetramethyl rhodamine methyl ester (TMRM) as previously described [20] in which the dye was present continuously at 50 nM. As a lipophilic cation, TMRM equilibrates between compartments in a Nernstian distribution, and so its concentration is a simple function of the potential differences between the cellular compartments. It is important to note that the TMRM fluorescence intensity is measured by excluding all background signal by ‘thresholding’ and then measuring the mean TMRM fluorescence intensity in the pixels containing mitochondria. Thus the signal is independent of mitochondrial mass and only reflects the dye concentration within the mitochondria.

### Assessing NADH Fluorescence as Mitochondrial Redox-index

Using a Zeiss UV–visible 510 CLSM, NADH autofluorescence was recorded from fibroblasts using excitation at 350 nm and emission measured between 435 and 485 nm. Fibroblasts were initially treated with the complex IV inhibitor NaCN to induce a maximal reduction. When a steady was obtained NaCN was washed out and replaced with standard recording buffer containing FCCP (1 µM) until the signals reached a steady state which now reflects the maximal oxidation state. All data were then normalized giving a set of data between ‘0’ (fully oxidised) and 1 (fully reduced) allowing us to obtain a value for the resting state which we called the ‘redox index’. Data underwent a further normalization setting the control as 1 to ease the graphic representation.

### Mdivi-1 Assay

Primary fibroblasts from *AnxA6* ^−/−^ and control mice were isolated and cultured on Matek dishes at 37°C and 5% CO_2_ for 24 to 72 h in standard DMEM buffer supplemented with 10% FCS, 100 U/ml penicillin, 100 µg/ml streptomycin, 2 mM L-glutamate (Invitrogen). Cells were washed with 37°C PBS and exposed to MitoTracker Green for 15–20 min at 37°C. MitoTracker Green (Molecular Probes) was first diluted 100x in DMSO and 1 or 2 µl of this solution were used per 2 ml of fresh 10% FCS DMEM phenol red-free media to incubate with cells. After incubation cells were washed 5–10 times in pre-warmed 25 mM HEPES buffer pH 7.4, 150 mM NaCl, and 2 ml of phenol red-free 10% FCS DMEM media supplemented with 25 mM HEPES buffer was added to the cells. Cells were transferred to the pre-warmed (37°C) Leica confocal microscope stage for live imaging of the mitochondrial network. Mdivi-1 (Enzo Life Sciences) was added to the media to a final concentration of 50 µM, mixed and incubated for up to 15 min. Mdivi-1 was first diluted to 10 mg/ml in DMSO and stored at -20°C. Mitochondrial network imaging commenced immediately after adding the Mdivi-1 and continued for up to 15 min. Control cells were exposed to 50 µM DMSO only.
